# Supplementary material for: Modeling glioblastoma heterogeneity as a dynamic network of cell states
Source: Mol Syst Biol. 2021 Sep 16;17(9):e10105. doi: 10.15252/msb.202010105 (PMC8444284; doi:10.15252/msb.202010105)
Supplement: Supplementary file 6 — Source Data for Figure 5 [file MSB-17-e10105-s004.zip › Figure5A_sourcedata/GSEA_3071/hallmarks_stateIV.GseaPreranked.1623742551702/HALLMARK_OXIDATIVE_PHOSPHORYLATION.html]

Details for gene set HALLMARK\_OXIDATIVE\_PHOSPHORYLATION[GSEA]

|  || Dataset | state53071 |
| Phenotype | NoPhenotypeAvailable |
| Upregulated in class | na\_pos |
| GeneSet | HALLMARK\_OXIDATIVE\_PHOSPHORYLATION |
| Enrichment Score (ES) | 0.10530017 |
| Normalized Enrichment Score (NES) | 0.5563919 |
| Nominal p-value | 0.9905437 |
| FDR q-value | 1.0 |
| FWER p-Value | 1.0 |
Table: GSEA Results Summary

  

Fig 1: Enrichment plot: HALLMARK\_OXIDATIVE\_PHOSPHORYLATION      
 Profile of the Running ES Score & Positions of GeneSet Members on the Rank Ordered List

  

| PROBE | GENE SYMBOL | GENE\_TITLE | RANK IN GENE LIST | RANK METRIC SCORE | RUNNING ES | CORE ENRICHMENT || 1 | PDP1 |  |  | 89 | 1.579 | 0.0243 | Yes |
| 2 | SLC25A4 |  |  | 352 | 0.973 | -0.0005 | Yes |
| 3 | ATP1B1 |  |  | 359 | 0.961 | 0.0235 | Yes |
| 4 | MAOB |  |  | 408 | 0.901 | 0.0378 | Yes |
| 5 | COX6C |  |  | 466 | 0.844 | 0.0490 | Yes |
| 6 | IDH1 |  |  | 469 | 0.842 | 0.0707 | Yes |
| 7 | GPX4 |  |  | 480 | 0.831 | 0.0905 | Yes |
| 8 | ATP5F1E |  |  | 634 | 0.706 | 0.0796 | Yes |
| 9 | NDUFA7 |  |  | 703 | 0.658 | 0.0838 | Yes |
| 10 | ATP6V0C |  |  | 737 | 0.639 | 0.0942 | Yes |
| 11 | NDUFA3 |  |  | 765 | 0.622 | 0.1053 | Yes |
| 12 | NDUFB4 |  |  | 999 | 0.522 | 0.0743 | No |
| 13 | UQCR11 |  |  | 1114 | 0.488 | 0.0652 | No |
| 14 | NDUFB5 |  |  | 1190 | 0.469 | 0.0631 | No |
| 15 | ACAA1 |  |  | 1193 | 0.468 | 0.0749 | No |
| 16 | COX17 |  |  | 1358 | 0.426 | 0.0546 | No |
| 17 | TIMM8B |  |  | 1407 | 0.415 | 0.0563 | No |
| 18 | ATP6AP1 |  |  | 1419 | 0.412 | 0.0650 | No |
| 19 | BAX |  |  | 1548 | 0.378 | 0.0503 | No |
| 20 | NDUFAB1 |  |  | 1628 | 0.363 | 0.0446 | No |
| 21 | DECR1 |  |  | 1648 | 0.359 | 0.0504 | No |
| 22 | ATP5F1D |  |  | 1817 | 0.328 | 0.0268 | No |
| 23 | NDUFA2 |  |  | 2047 | 0.290 | -0.0096 | No |
| 24 | ATP6V1G1 |  |  | 2148 | 0.277 | -0.0215 | No |
| 25 | COX6B1 |  |  | 2309 | 0.253 | -0.0456 | No |
| 26 | NDUFB7 |  |  | 2329 | 0.250 | -0.0427 | No |
| 27 | MFN2 |  |  | 2371 | -0.255 | -0.0439 | No |
| 28 | IMMT |  |  | 2380 | -0.256 | -0.0387 | No |
| 29 | ACAA2 |  |  | 2390 | -0.257 | -0.0337 | No |
| 30 | CYB5R3 |  |  | 2469 | -0.266 | -0.0417 | No |
| 31 | ATP5MC3 |  |  | 2620 | -0.285 | -0.0630 | No |
| 32 | AFG3L2 |  |  | 2636 | -0.288 | -0.0584 | No |
| 33 | IDH3B |  |  | 2668 | -0.291 | -0.0567 | No |
| 34 | MRPS11 |  |  | 2685 | -0.293 | -0.0521 | No |
| 35 | NDUFC2 |  |  | 2716 | -0.296 | -0.0501 | No |
| 36 | MRPL11 |  |  | 2735 | -0.298 | -0.0457 | No |
| 37 | CYC1 |  |  | 2742 | -0.299 | -0.0391 | No |
| 38 | NDUFA9 |  |  | 2878 | -0.317 | -0.0567 | No |
| 39 | CASP7 |  |  | 2896 | -0.319 | -0.0516 | No |
| 40 | OGDH |  |  | 2941 | -0.324 | -0.0515 | No |
| 41 | TIMM10 |  |  | 2963 | -0.328 | -0.0470 | No |
| 42 | TIMM17A |  |  | 2986 | -0.331 | -0.0426 | No |
| 43 | SLC25A3 |  |  | 3087 | -0.343 | -0.0528 | No |
| 44 | ATP6V0B |  |  | 3115 | -0.349 | -0.0488 | No |
| 45 | SDHD |  |  | 3136 | -0.352 | -0.0434 | No |
| 46 | ETFA |  |  | 3215 | -0.363 | -0.0489 | No |
| 47 | MRPL15 |  |  | 3217 | -0.363 | -0.0396 | No |
| 48 | PDHA1 |  |  | 3244 | -0.367 | -0.0350 | No |
| 49 | FXN |  |  | 3259 | -0.369 | -0.0280 | No |
| 50 | ACAT1 |  |  | 3282 | -0.372 | -0.0225 | No |
| 51 | CYB5A |  |  | 3418 | -0.394 | -0.0381 | No |
| 52 | ATP6V1D |  |  | 3441 | -0.396 | -0.0319 | No |
| 53 | HCCS |  |  | 3470 | -0.403 | -0.0267 | No |
| 54 | NDUFA6 |  |  | 3511 | -0.412 | -0.0236 | No |
| 55 | ATP5F1C |  |  | 3628 | -0.433 | -0.0345 | No |
| 56 | MRPL35 |  |  | 3655 | -0.436 | -0.0281 | No |
| 57 | VDAC1 |  |  | 3662 | -0.437 | -0.0178 | No |
| 58 | CPT1A |  |  | 3665 | -0.437 | -0.0067 | No |
| 59 | OAT |  |  | 3714 | -0.448 | -0.0042 | No |
| 60 | GRPEL1 |  |  | 3721 | -0.449 | 0.0064 | No |
| 61 | MDH2 |  |  | 3798 | -0.466 | 0.0040 | No |
| 62 | OXA1L |  |  | 3810 | -0.469 | 0.0142 | No |
| 63 | SDHB |  |  | 3887 | -0.484 | 0.0123 | No |
| 64 | ATP5PB |  |  | 3908 | -0.489 | 0.0212 | No |
| 65 | MGST3 |  |  | 3917 | -0.491 | 0.0325 | No |
| 66 | MRPS30 |  |  | 3937 | -0.494 | 0.0418 | No |
| 67 | ATP5MC1 |  |  | 4020 | -0.518 | 0.0397 | No |
| 68 | CYCS |  |  | 4074 | -0.532 | 0.0434 | No |
| 69 | COX5A |  |  | 4172 | -0.560 | 0.0395 | No |
| 70 | FH |  |  | 4182 | -0.562 | 0.0525 | No |
| 71 | SLC25A5 |  |  | 4296 | -0.597 | 0.0464 | No |
| 72 | TIMM9 |  |  | 4300 | -0.597 | 0.0615 | No |
| 73 | HSPA9 |  |  | 4410 | -0.639 | 0.0573 | No |
| 74 | NQO2 |  |  | 4532 | -0.697 | 0.0524 | No |
| 75 | LDHA |  |  | 4574 | -0.722 | 0.0634 | No |
| 76 | MRPS15 |  |  | 4739 | -0.821 | 0.0534 | No |
| 77 | GLUD1 |  |  | 4786 | -0.849 | 0.0668 | No |
| 78 | IDH2 |  |  | 5022 | -1.133 | 0.0514 | No |
Table: GSEA details [plain text format]

  

Fig 2: HALLMARK\_OXIDATIVE\_PHOSPHORYLATION: Random ES distribution      
 Gene set null distribution of ES for **HALLMARK\_OXIDATIVE\_PHOSPHORYLATION**
